# Establishing of mouse oral carcinoma cell lines derived from transgenic mice and their use as syngeneic tumorigenesis models

**DOI:** 10.1186/s12885-019-5486-7

**Published:** 2019-03-29

**Authors:** Yi-Fen Chen, Chung-Ji Liu, Li-Han Lin, Chung-Hsien Chou, Li-Yin Yeh, Shu-Chun Lin, Kuo-Wei Chang

**Affiliations:** 1 Institute of Oral Biology, School of Dentistry, National Yang-Ming University, No. 155, Li-Nong St., Section 2, Beitou District, Taipei, 112 Taiwan; 2 Department of Dentistry, School of Dentistry, National Yang-Ming University, No. 155, Li-Nong St., Section 2, Beitou District, Taipei, 112 Taiwan; 3 Department of Dentistry, MacKay Memorial Hospital, No. 45, Minsheng Rd., Tamsui District, New Taipei City, 251 Taiwan; 4 Department of Stomatology, Taipei Veterans General Hospital, No.201, Sec. 2, Shipai Rd., Beitou District, Taipei, 112 Taiwan

**Keywords:** Mouse, microRNA, *miR-211*, Oral carcinoma, Tongue

## Abstract

**Background:**

The survival of OSCC patient needs to be further improved. *miR-211* is oncogenic in OSCC and its upregulation is associated with tumor progression and poor patient survival. K14-EGFP-*miR-211* transgenic mice also exhibit augmented potential for OSCC induction.

**Methods:**

Four murine OSCC cell lines, designated MOC-L1 to MOC-L4, are established from tongue tumors induced by 4-nitroquinoline 1-oxide using the K14-EGFP-*miR-211* transgenic mouse model. The genetic disruption, in vitro oncogenicity, and the eligibilities of tumorigenesis and metastasis of the cell lines are analyzed.

**Results:**

All cell lines show green fluorescence and express a range of epithelial markers. The MOC-L1, MOC-L2 and MOC-L3 cells carry missense mutations in the DNA binding domain of the *p53* gene. MOC-L1 exhibits a high level of epithelial-mesenchymal transition and has the aggressive characteristics associated with this. MOC-L1 and MOC-L2 are clonogenic in vitro as well as being tumorigenic when implanted into the dermis or tongue of syngeneic recipients. Nonetheless, only MOC-L1 exhibits immense potential for local regional and distal metastasis. Since the expression of *miR-196b* in MOC-L1 xenografts is drastically decreased on cisplatin treatment, it would seem that targeting of *miR-196b* might facilitate tumor abrogation.

**Conclusions:**

As cell lines established in this study originated from the C57BL/6 mouse, the strain most suitable for transgenic engineering, exploring the interplay of these OSCC cells with other genetically modified cells in immune-competent mice would provide important insights into OSCC pathogenesis.

**Electronic supplementary material:**

The online version of this article (10.1186/s12885-019-5486-7) contains supplementary material, which is available to authorized users.

## Background

Head and neck squamous cell carcinoma (HNSCC), including oral squamous cell carcinoma (OSCC), is a common malignancy worldwide. The OSCC patients five-year survival rate is generally around 50% [1]. Despite that, the fact that tissue assay and cell line analysis have yielded abundant findings that have advanced significantly the diagnosis and treatment of OSCC, the survival of OSCC patients has not been improved profoundly in the last few decades. Human OSCC cell lines have contributed significantly helping to elucidate the mechanisms associated with OSCC pathogenesis. However, the use of immunocompromised murine systems, which has allowed the xenografic growth of human OSCC cell lines, has not been sufficient when trying to address the in vivo neoplastic process and treatment responses; this is due to the lack of an immune microenvironment [2]. The establishment of murine OSCC cell lines and their subsequent preclinical assessment using immunocompetent recipients would be an important step to facilitating the use of a translational approach.

Several mouse OSCC cell lines have been established using C3H, BALB/c and DBA/2 mouse strains, and these have yielded profound pathogenic insights into OSCC [3–5]. In this context, the mouse strain C57BL/6 is the one that is most commonly used for gene engineering; this has led to rapid progress in terms of genetic engineering mouse models. Thus, the induction of OSCC using a combination of a genetically engineered background and chemical carcinogen treatment will open up a door to understanding the exogenous and endogenous factors that contribute to OSCC in C57BL/6 mice [2]. miRNA disruption plays an important role in OSCC pathogenesis [6–17]. We have established *miR-31* and *miR-211* transgenic (Tg) mouse lines that have these transgenes overexpressed in the mouse basal keratinocytes [7, 8, 18]. These mice show higher frequency and faster OSCC tumor induction following 4-nitroquinoline 1-oxide (4NQO) treatment [7, 8, 18, 19]. By means of these models, we have uncovered new suppressors that are targeted by these oncogenic miRNAs and unraveled the involvement of DNA defects and the enrichment of oxidative stress in OSCC progression. In addition, due to the rapid tumor induction and fluorescent tumor labeling in these mice, the models have been used to enable new developments in image diagnosis [20].

Xenotransplantation requires a relatively shorter time period to obtain a full-blown tumor than chemical treatment [2]. In addition, tumor xenografts have more homogeneous characteristics compared to chemically induced lesions. Xenografts of human cancer cells into immuno-compromised mice have helped with the functional elucidation of tumor growth and its interception. However, being able to carry out orthotopic xenotransplantation of mouse OSCC cells into immunocompetent syngeneic mice would help us to obtain a better and a more comprehensive understanding of tumor complexity, which in part is due to the presence of a relevant tumor microenvironment and appropriate immuno-modulation [21]. This study establishes, for the first time, four murine OSCC cells lines; these were obtained from 4NQO treated transgenic mice. The genetic disruption and aggressiveness of these cell lines, their tumorigenicity, their ability to bring about both local regional metastasis and distal metastasis in C57BL/6 syngeneic mice are defined in the present study. These cell lines and the linked immunocompetent animal model that we have established will facilitate the investigation of therapies that can be used to treat OSCC.

## Methods

### Induction of OSCC from K14-EGFP-*miR-211* Tg mice

*miR-211* is an oncogenic miRNA associated with OSCC [7, 8, 14, 15]. The K14-EGFP-*miR-211* Tg mouse has been established in C57BL/6 previously using the murine pri-*miR-211* sequence tagged with a green fluorescence protein (GFP) [7]. For OSCC induction, 100 μg/ml of 4NQO was added to the drinking water of 6–8 week-old mice for 16 weeks. Mice were sacrificed at a time point when their body weight loss was > 1/3, when tumors had begun to interfere with their food uptake, when they showed weakness, or when at a specific endpoint [7, 11, 18].

### Establishing the MOC-L cell lines

The human OSCC SAS cell line was obtained from the JCRB cell bank, while the FaDu and 293 T ell lines were obtained from the ATCC cell bank. Human cell lines were authenticated by short tandem repeat analysis. Mouse OSCC lesions on the dorsal tongue surface were dissected and introduced into the primary culture. The cells were maintained in complete DMEM medium (Thermo Scientific, Waltham, MA) containing 10% FBS (Biological Industries), 2 mM L-glutamine (Biological Industries) and 1% pen-strep-ampho (Biological Industries). The cultivation conditions are described in Additional file 1: Table S1. Defined keratinocyte serum-free medium (KSFM; Thermo Scientific) was used during culture to limit the growth of cells other than keratinocytes. A region of the *PTGER2* gene that shows high homology between the human and mouse sequences was analyzed using species-specific PCR (primers listed in Additional file 1: Table S2) in order to confirm the origin of the cell lines [22]. Stable cell subclones expressing luciferase were achieved by transfecting the pGL4.5 Luc plasmid (Promega, Madison, WI) into a given cell line and this was then followed by selection. All reagents were purchased from Sigma-Aldrich (St Louise, MO) unless specified otherwise.

### Phenotypic assays

Cells were seeded into 96-well plates at a density of 2500 cells per well, and the cell proliferation over the following 4 days was then analyzed by MTT assay. The cell growth curves of the various cell lines were plotted in order to calculate the population doubling time of each cell line. The cell migration and invasion assays were performed using 0.8 μm 24-well Transwell chambers (Merck Millipore, Billerica, MA). For the migration assays, cells were collected and seeded into the upper chamber of a Transwell at a density of 1 × 10^5^ cells per well. For the invasion assays, 50 μl of 10% Matrigel (BD Biosciences, San Jose, CA) was used to coat each Transwell chamber and then 2 × 10^5^ cells were seeded onto the Matrigel-coated Transwell. After incubating at 37 °C for 48 h, the Transwell membranes were fixed and stained with 10 μg/ml Hoechst 33258 for 10 min. Images of the migrated or invading cells were captured using a fluorescence microscope. The cell numbers in each picture were counted and then normalized to allow the fold-change to be calculated. For colony formation assays, cells were seeded into 6-well plates at a density of 100 cells per well to allow for colony formation. Ten days later, the colonies were washed twice with PBS, fixed with ethanol and stained with 0.05% crystal violet. Colonies were counted by microscopy [7, 17].

### Western blot analysis

Western blot analysis followed the approach of previous protocols [7, 17]. The primary antibodies are listed in Additional file 1: Table S3. Signals were revealed by Western Lightening Chemiluminescence Reagent Plus kit (Thermo Scientific) and detected using a FUJIFILM LAS-4000 mini luminescent image analyzer (GE Life Sciences, Piscataway, NJ).

### RNA extraction and PCR analysis

Cells were collected in TriPure Isolation Reagent (Roche, Basel, Switzerland) and disrupted using 1-bromo-3-chloropropane. The lysate was collected and the upper aqueous phase, which contains the RNA, was moved. The aqueous layer was next mixed thoroughly with isopropanol and then spun at maximum speed to pellet the precipitated RNA. The resulting pellet was washed and then dissolved in nuclease-free water. The various miRNA TaqMan® assay (Thermo Scientific) probes used in this study are described in Additional file 1: Table S4. The threshold cycle (C_t_) method was used. The resulting information was analyzed using the –ΔΔC_t_ method and values calculated relative to internal controls [17]. A mutation of the *p53* gene was also analyzed using PCR analysis (primers listed in Additional file 1: Table S2).

### Immunohistochemistry (IHC)

Formalin-fixed paraffin tissue samples were sliced into 5 μm sections, deparaffinized with xylene, rehydrated, and subjected to antigen retrieval solution. IHC was carried out using the UltraVision Quanto Detection System IHC staining kit (Thermo Scientific) by following the manufactured protocol. The anti-GFP antibody is described in Additional file 1: Table S3. The tissue sections exhibiting faint and/or scattered GFP immunoreactivity were scored +, while sections exhibiting dense and/or extensive GFP immunoreactivity were scored ++.

### Xenograft transplantation

A total of 5 × 10^6^ cells were injected into the flank of each non-obese diabetic CB17-Prkdc^scid^/NcrCrlBltw (NOD-SCID) mouse (National Laboratory Animal Center, Taipei, Taiwan). The cells used were mixed 1:1 with Matrigel (BD Biosciences), which gave a total volume of 200 μl. These mice were sacrificed during the 5th week. A total of 1 × 10^7^ cells were injected into the flank of each C57BL/6 mouse (BioLASCO, Taipei, Taiwan). These cells were also mixed 1:1 with Matrigel (BD Biosciences) to give a total volume of 200 μl. However, these mice were sacrificed during the 2nd week.

For the orthotopic xenografts, 5 × 10^6^ cells in a total volume of 100 μl were injected into the central portion of the tongue of C57BL/6 mice (BioLASCO). These mice were sacrificed between the 3rd and 6th week in order to detect the rate of growth of the primary tumor and the presence of neck metastasis. In addition, nude mice (National Laboratory Animal Center) were also used to assess the neck metastasis capability and this was done by injecting 5 × 10^6^ of cells (in 100 μl volume) into the central portion of the tongue. These mice were sacrificed between the 7th and 9th week. The tongue tumors and the neck tissues from the mice were isolated to allow histopathological and IHC evaluation.

For the in vivo real-time tumor imaging, 5 × 10^6^ cells were injected into the tongue central portion of C57BL/6 mice (BioLASCO). An IVIS Spectrum system (Caliper Life Science, Hopkinton, MA) was used for detecting tumor growth and this was done once a week. The mice were sacrificed during the 8th week. Tongue tumors and neck tissue were isolated for histopathological evaluation.

For the distant metastasis assay, 1 × 10^6^ cells were injected intravenously into C57BL/6 mice (BioLASCO). These mice were sacrificed when body weight loss > 1/3 or between the 7th and the 9th week. An autopsy was performed on each mouse in order to evaluate the metastasis and the presence of tumor foci in each animal’s various organs.

To test the therapeutic efficacy of cisplatin (CDDP), cells that had been mixed 2:1 with Matrigel (BD Biosciences) to give a total volume of 150 μl were injected into C57BL/6 mice (BioLASCO) subcutaneously. When tumor volume had reached about 0.2 mm^3^ during the 3rd week after tumor injection, 2 mg/kg CDDP dissolved in 0.9% saline was intraperitoneally injected every other day for four weeks. In parallel, the control group received an equal volume injection of 0.9% saline [23, 24]. The tumor sizes were measured every week.

The primary tumors, the neck regions of the mice and the lungs of the mice were photographed using Illumatool Bright Light System (LT-9500; TLS, Sarasota, FL) in order to visualize the primary tumors and the various metastasis lesions that were exhibiting green fluorescence. All mice were sacrificed by CO_2_ inhalation at the end point of the experiment. All resected tissues were subjected to histopathological evaluation. The tumor volumes were calculated using the formula = 0.5x*a*x*b*^2^, the parameters having been measured at a micro-scale under light microscopy or using a gauge. For this calculation, *a* was the longest diameter and *b* was the shortest diameter. All animal studies were carried out followed the guideline of National Yang-Ming University Institutional Animal Care and Use Committee (IACUC).

### Statistical analyses

The results are presented as means ± SE. Mann-Whitney tests, t-tests, two-way ANOVA tests, correlation analysis, and Kaplan-Meier survival analysis were used to compare the differences between the various groups of results. A *p*-value of less than 0.05 was considered statistically significant.

## Results

### Establishing and characterizing the mouse oral cancer cell lines

**Fig. 1:**
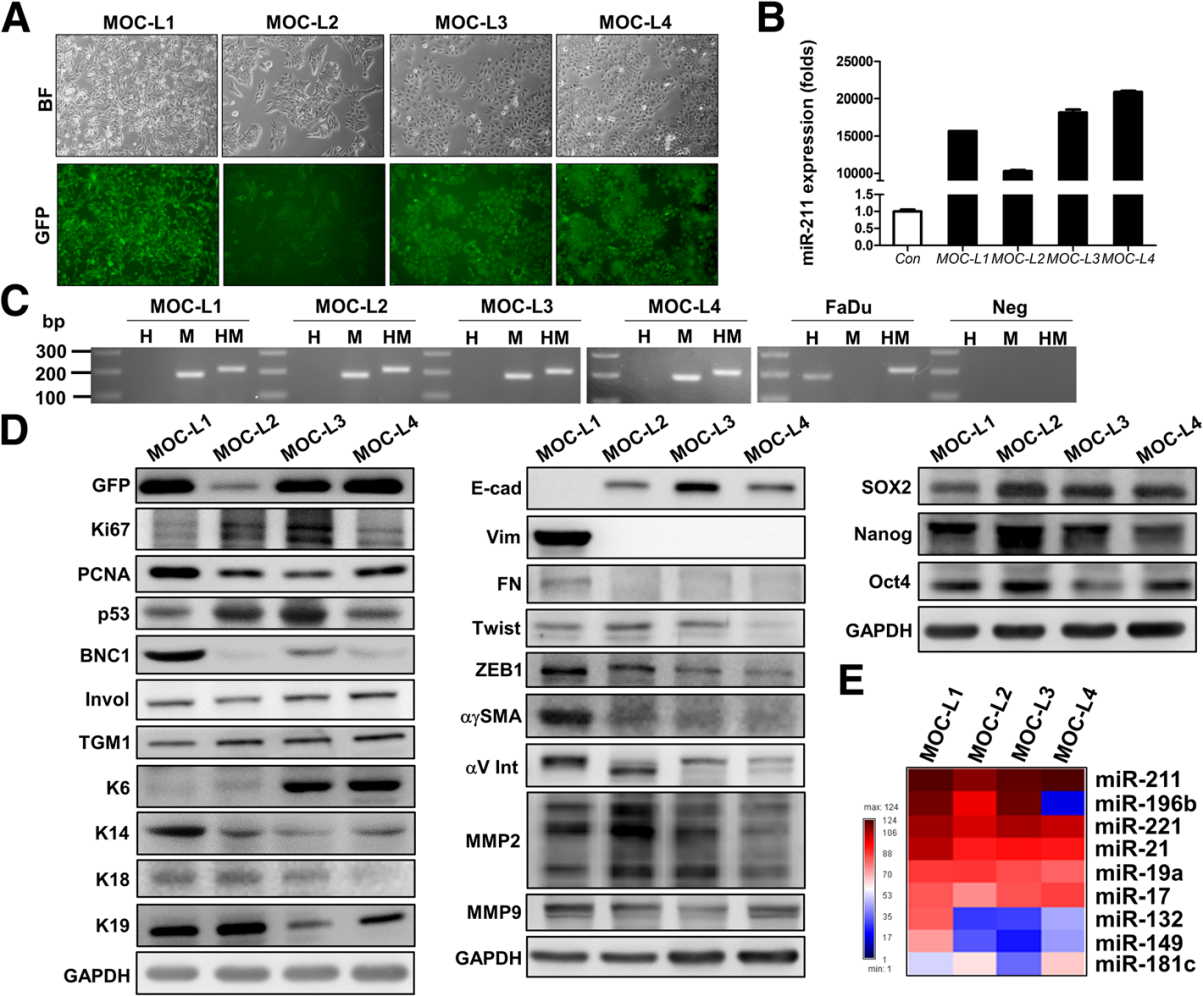
Establishing the cell lines and their characterization. **a** Cell morphology and fluorescence image of MOC-L1 to MOC-L4. × 100. Upper, bright field inverted microscopy; Lower, fluorescence microscopy. **b** Quantitative PCR analysis. This shows the high level of *miR-211* expression in these four cell lines. **c** Typing of the cell origins by PCR analysis. H, human; M, mouse; HM, both human and mouse. The results show that these cell lines originated from mouse. Neg, negative control. The other lanes in gels are molecular weight markers. **d** Western blot analysis of gene expression across the various cell lines. Left, proliferation and differentiation genes; Middle, EMT markers; right, stemness markers. **e** The expression profile algorithm of the selected miRNAs in MOC cell lines relative to the SAS cell line. The miRNAs analyzed are shown in Additional file 1: Figure S1a

Using the K14-EGFP*-miR-211* Tg mice, intake of drinking water containing 4NQO resulted in oral carcinogenesis, especially of sites on the tongue. Upon sacrifice of the animals, exophytic fluorescent masses were found to be present on the dorsal tongue surface, and those with a size greater than 0.3 cm were dissected. The main parts of the tumor masses were washed, minced into smaller pieces and then dissociated in order to release tumor cells. The remaining tissues were embedded to allow histopathological evaluation and the confirmation of the malignancy. The dissociated cells were cultivated in 10% DMEM. Non-keratinocytes that did not show fluorescence were eliminated by cultivation in KSFM for three weeks. Four individual primary cells survived and showed continuous growth exceeding 100 days; these became our four cell lines. These OSCC cell lines appeared polygonal, ovoid or spindle-shaped, and they exhibited green fluorescence were then designated as the Mouse Oral Carcinoma - Line (MOC-L) series, which consisted of MOC-L1, MOC-L2, MOC-L3, and MOC-L4 (Fig. 1a). High *miR-211* expression was detected in all of these cell lines, which assured their transgenic genetic background (Fig. 1b). PCR genotyping using *PTGER2* species-specific primers validated their mouse originality (Fig. 1c). The expression of the GFP tag was confirmed by Western blot analysis (Fig. 1d). The low level of GFP expression by MOC-L2 is consistent with its lower fluorescence and lower level of *miR-211* expression. MOC-L2 and MOC-L3 showed high Ki67 and p53 expression, while MOC-L1 had the highest level of PCNA expression. All cell lines expressed both involucrin and TGM1 and this further assured their epithelial origin. MOC-L1 exhibited intensive expression of various basal keratinocyte markers, including BCN1, K14, and K19, while MOC-L3 and MOC-L4 showed strong K6 expression, this protein usually expressed in the differentiation layer of squamous epithelium. It was interesting to note the presence of E-cadherin (E-cad) expression and the absence of vimentin expression in the MOC-L2, MOC-L3, and MOC-L4 cells. However, MOC-L1 did exhibit strong epithelial-mesenchymal transition (EMT) characteristics in that it showed a lack of E-cad expression and the presence of strong vimentin expression (Fig. 1d). Moreover, the expression levels of other mesenchymal regulators, such as fibronectin, ZEB1, and αγSMA were also stronger in MOC-L1 relative to the other cell lines. All of the cell lines expressed stemness markers, while MOC-L2 showing the strongest expression of SOX2, Nanog, and OCT4 among them (Fig. 1d). In addition, several oncogenic miRNAs such as *miR-17*, *miR-19*a, *miR-21,* and *miR-221* were found to be highly expressed in these MOC-L cell lines (Fig. 1e and Additional file 1: Figure S1a). Finally, *miR-196b* was highly expressed in MOC-L1, MOC-L2 and MOC-L3, but the expression of *miR-196b* in MOC-L4 was almost absent.

### Phenotypic analysis of the MOC-L cell lines

**Fig. 2:**
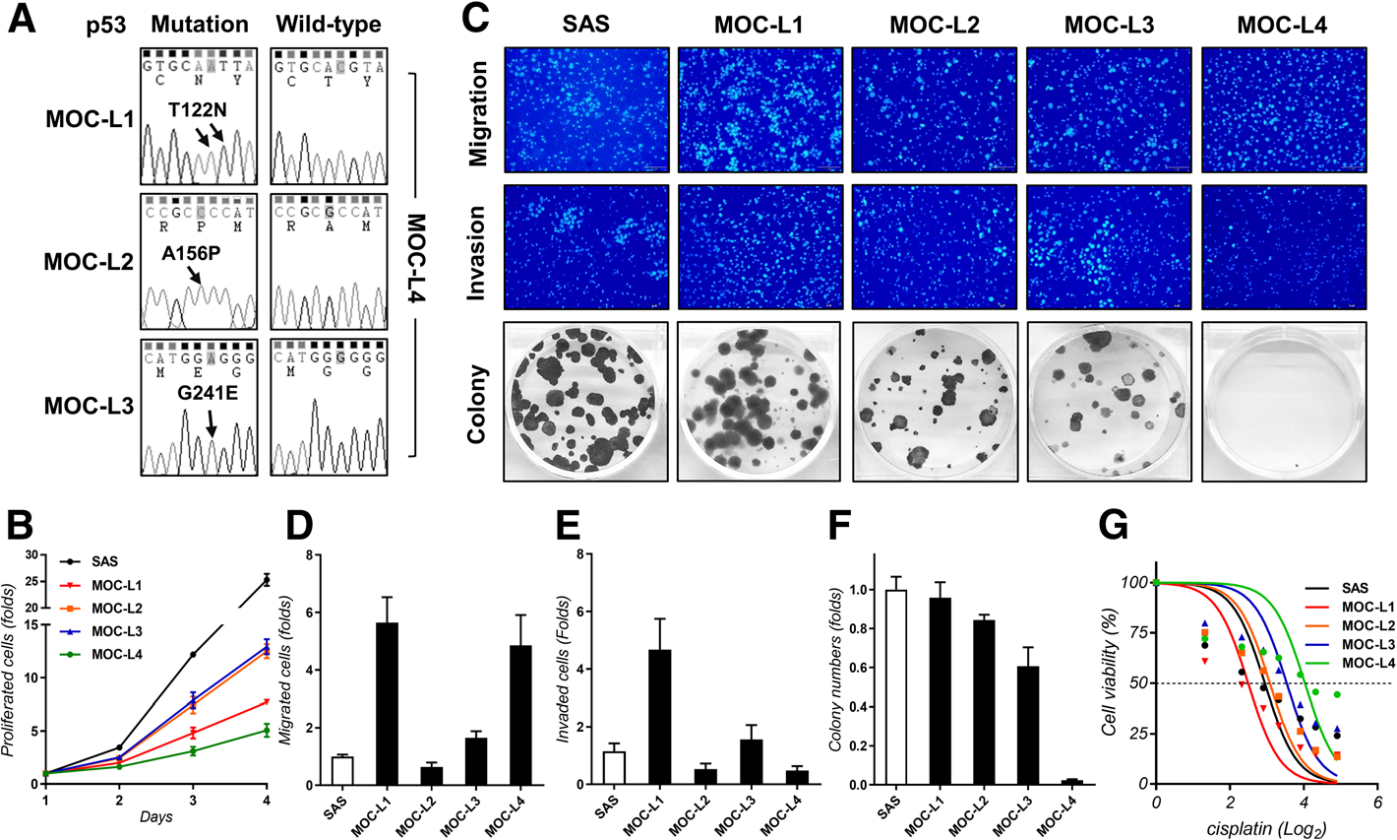
Analysis of *p53* mutation and phenotype. **a** *p53* sequencing. It reveals the presence of *p53* missense mutations in the genomic DNA of MOC-L1, MOC-L2, and MOC-L3. The right panels show the sequencing of the MOC-L4 cell line demonstrating the absence of a *p53* mutation. Arrows indicate the mutated nucleotides in the other cell lines. **b** Proliferation assay. **c** Representative results showing the migration (Upper, × 100), invasion (Middle, × 100) and colony formation assays (Lower). **d-f** Quantification of migration, invasion and colony formation capability relative to the SAS cell line. **g** Dose-response curve of the cell lines following 0, 2.5, 5, 7.5, 10, 15, 20, and 30 μM CDDP treatment for 24 h

cDNAs isolated from the four cell lines were amplified and sequenced in order to determine the presence of the T122 N, A156P and G241E mutations of the *p53* gene in the MOC-L1, MOC-L2 and MOC-L3 cell lines; these mutations correspond to human *p53* codon 125, 159 and 244, respectively. The nucleotide changes were found to be ACG to AAT in MOC-L1, GCC to CCC in MOC-L2 and GGG to GAG in MOC-L3 (Fig. 2a). MOC-L4 was the least aggressive cell line which had the lowest proliferation rate (Fig. 2b), invasion ability (Fig. 2c, e), and colony formation ability (Fig. 2c, f). MOC-L4 also had high migration competence and the highest tolerance to CDDP treatment (Fig. 2d, g). By way of contrast, MOC-L1 was the most aggressive cell line in terms of invasion and colony formation capabilities. MOC-L2 and MOC-L3 were similar in their modest oncogenic phenotypes and their sensitivity to CDDP (Fig. 2b-g). The population doubling time of MOC-L1, MOC-L2, MOC-L3 and MOC-L4 were calculated to be 24.7, 20.4, 20.3 and 29.3 h, respectively.

### Xenotransplantation of the MOC-L cell lines

**Fig. 3:**
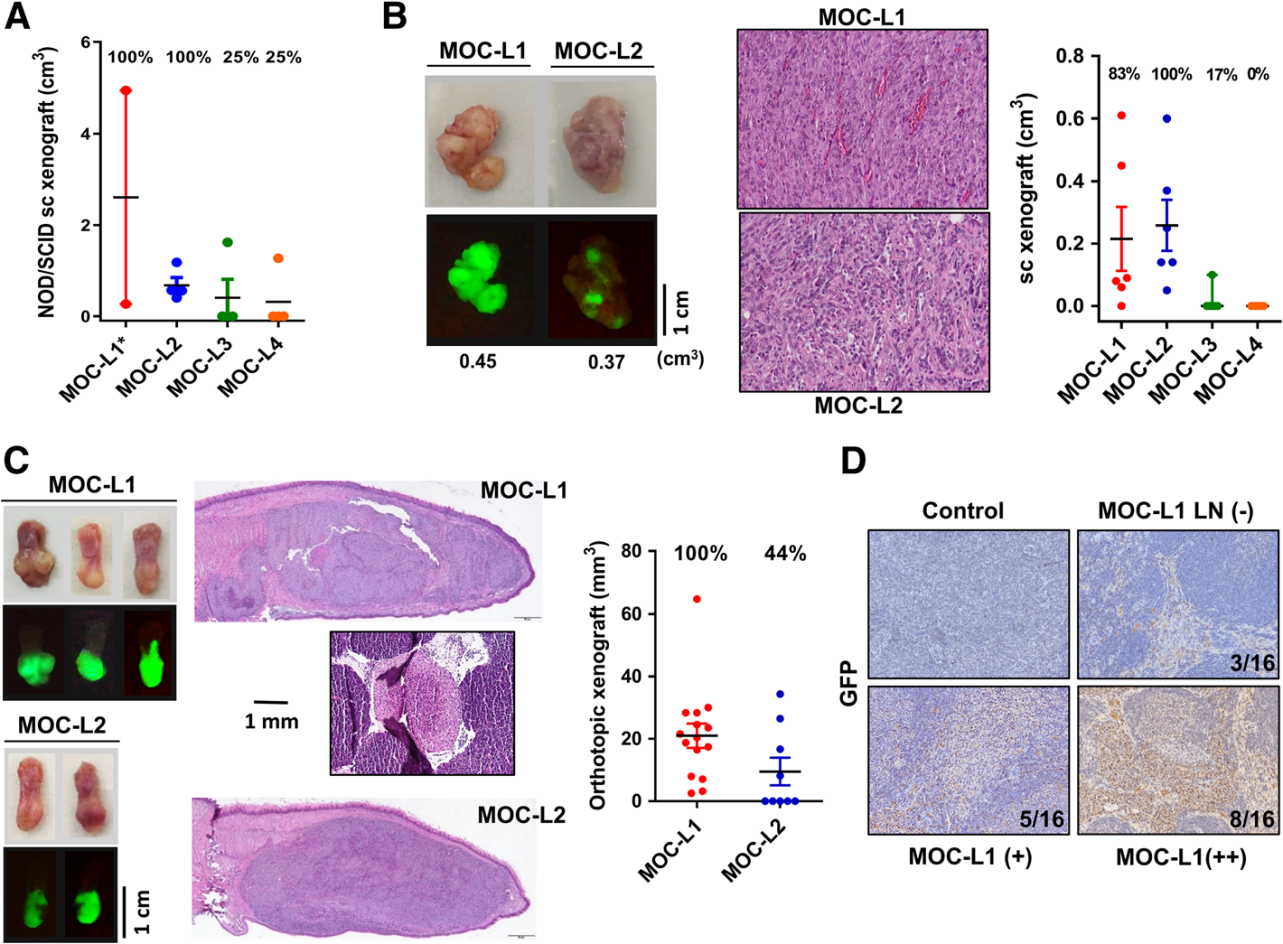
Xenotransplantation of cell lines. **a** Subcutaneous xenografic induction of cells admixed with Matrigel in NOD-SCID mice. Tumors are harvested 2 weeks after inoculation. * denotes that two recipients who show rapid tumor growth and die during the first two weeks; these are excluded for the analysis. The tumorigenicity of MOC-L1 and MOC-L2 is greater than that of MOC-L3 and MOC-L4. **b** Subcutaneous xenografic induction of cells in C57BL/6 mice. Left, gross pictures (Upper) and fluorescence images (Lower) of the resected tumors. Middle, histopathological pictures of the tumors. X200. Right, quantification. MOC-L1 and MOC-L2 exhibit syngeneic tumor induction, while MOC-L3 and MOC-L4 are nearly non-tumorigenic. **c** Orthotopic xenografic induction of MOC-L1 and MOC-L2 cells in C57BL/6 mice. Left, gross pictures (Upper) and fluorescence images (Lower) of the resected tongues. Middle, histopathological sections of the tongues revealing the presence of the tumors within each tongue. Note the presence of a positive neck node in a mouse implanted with MOC-L1 cells. Right, quantification of the tongue tumors. X100. **d** GFP immunohistochemistry of the neck lymph nodes of nude mice that had undergone orthotopic MOC-L1 xenotransplantation. -, negative, +, weak positive; ++, strong positive. Control, mice without xenotransplantation

To test the tumorigenic potential of the four cell lines, we first performed xenotransplantation of a cell/Matrigel mixture into the flank of NOD-SCID mice. Over a five-week-period, among the four mice that had received MOC-L1 injection and exhibited tumor growth, two died unexpectedly due to vigorous tumor growth and weakness. MOC-L1 seems to be rather deadly to NOD-SCID mice. MOC-L2 was also quite tumorigenic, while the tumorigenicities of MOC-L3 and MOC-L4 were relatively low (Fig. 3a). The MOC-L1 and MOC-L2 tumors in the NOD-SCID mice were dissected and re-cultured to achieve cells adapted to both in vitro and in vivo environments for subsequent experiments. In the syngeneic mice, MOC-L1 and MOC-L2 also grew well in the dermis, but the subcutaneous growth potentials of MOC-L3 and MOC-L4 were nearly absent (Fig. 3b). Both MOC-L1 and MOC-L2 grew continuously when they were seeded into the tongue of syngeneic mice. The orthotopic xenografic growth potential of MOC-L1 exceeded that of MOC-L2. In addition, neck lymph node metastasis was occasionally seen, but only in mice harboring MOC-L1 (Fig. 3c). To ascertain that MOC-L1 is able to produce neck metastasis, the orthotopic xenotransplantation of MOC-L1 was carried out in nude mice. The histopathological sections of the dissected tissues revealed the presence of GFP-positive polygonal cells in more than 80% of the lymph nodes (Fig. 3d). GFP immunoreactivity was not detectable in the neck lymph nodes of any of the control mice. These results indicated that both MOC-L1 and MOC-L2 are tumorigenic in syngeneic mice and the former also possess a competence for neck dissemination.

### Distant metastasis of the MOC-L1 cell line

**Fig. 4:**
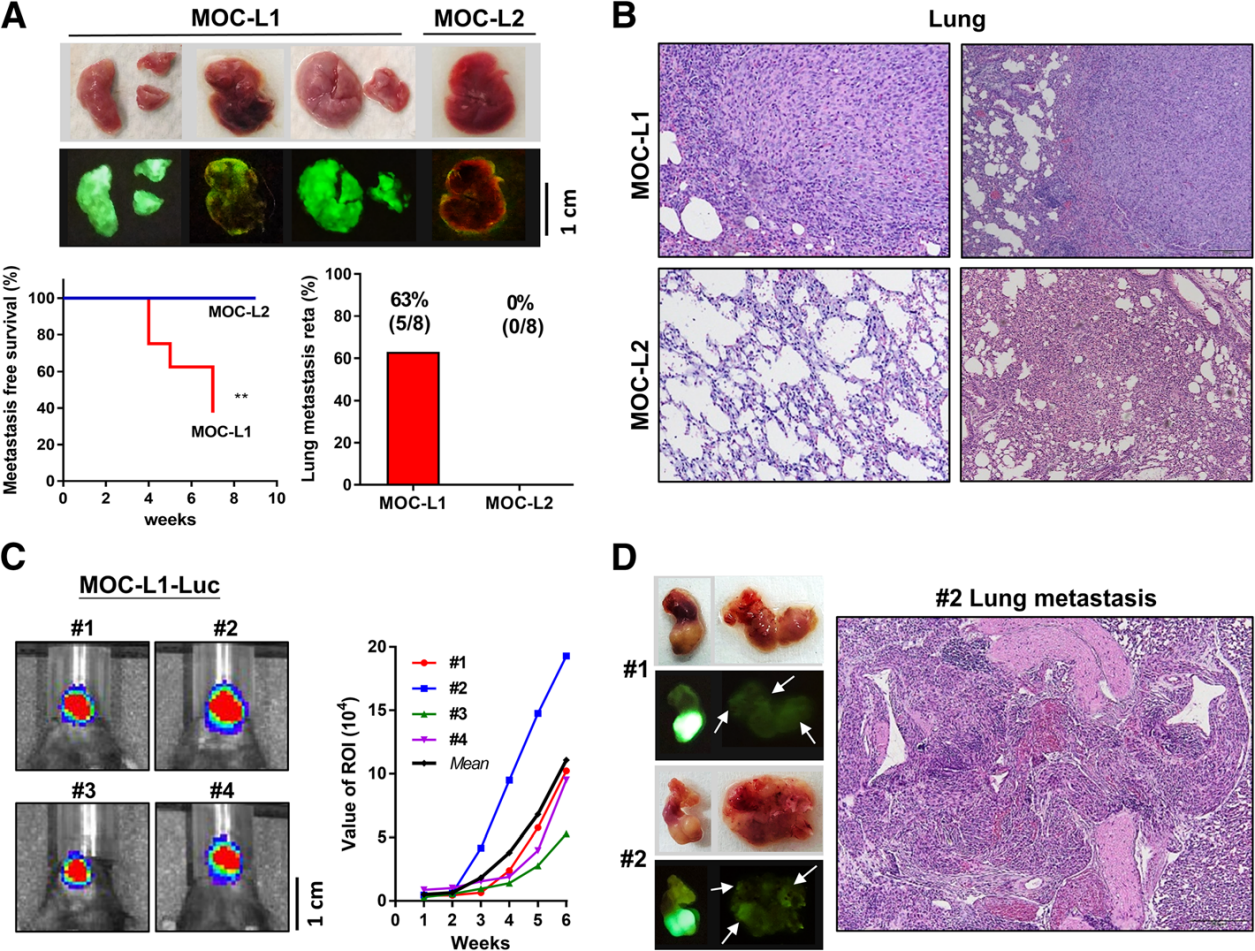
High metastasis rate of the MOC-L1 cell line. **a** Tail vein injection of MOC-L1 and MOC-L2 cells into C57BL/6 mice. The survival status over 7 weeks (for MOC-L1) and 9 weeks (for MOC-L2) and an examination of the resected lungs. Upper, representative gross pictures. Middle, fluorescence images of the lungs. Note the intense and diffuse fluorescence in the lungs of the mice that have received a MOC-L1 cell injection, but the absence of these findings in the mice who have received the injection of MOC-L2 cells. Lower Left, mice who received the MOC-L1 injection exhibit a significantly worse metastatic-free survival. **, *p* < 0.01 **b** Representative tissue sections of the lungs. Metastatic lesions are evident in the lungs of mice that had received an injection of MOC-L1 cells. The sections of lungs received the injection of MOC-L2 cells show normal structure or chronic inflammatory states. X100. The quantitation of lung metastasis is depicted in the Lower Right panel in **a**. Only mice that have received the MOC-L1 injection show lung metastasis. **c** Orthotopic xenotransplantation of MOC-L1-Luc cells into four C57BL/6 mouse hosts (#1 - #4). Left, Detection of luciferase activity at week 6. Right, the real-time luciferase measurements that illustrate the growth potential of the MOC-L1-Luc cell subclone in their hosts. The tumor in mouse #2 exhibits the most conspicuous growth. **d** Detection of green fluorescence. Left, representative harvested tongue and neck tissue samples. Upper, gross images; Lower, fluorescence images. Note the faint fluorescent spots indicated by arrows in the neck tissues. Right, a representative histopathological section of lung. X200. This reveals the metastasis of cells to the hilar region of the lung and adjacent tissues

The MOC-L1 and MOC-L2 cell lines were injected into the tail vein of syngeneic mice to determine their potential of distal metastasis. During the follow-up period of 7 to 9 weeks, 63% the MOC-L1 injected mice were found to have diffused or scatter tumor nodules and green fluorescence in the lungs, but neither nodules nor green fluorescence was detected in the MOC-L2 injected mice (Fig. 4a). Histopathological examination confirmed the existence of lung metastasis in the MOC-L1 mice only. Tissue sections of the lungs of the mice that received MOC-L2 injections showed parenchymal inflammation, fibrosis or a normal lung tissue architecture without tumor involvement (Fig. 4b).

To validate the possibility of real-time monitoring of tumor growth, we also established a MOC-L1-Luc cell subclone. This subclone has been successfully used to create growth curves of orthotopic xenografts over an 8 weeks period in syngeneic mice (Fig. 4c). We also noted that the relatively lower growth potential of this cell subclone in comparison with its parental cells. Nevertheless, the neck metastasis potential of this subclone was confirmed by the presence of faint green fluorescence spots in neck tissue and lung metastasis capability was confirmed by histopathological examination (Fig. 4d). The induction of orthotopic xenografts using the MOC-L2-Luc cell subclone was not successful.

### CDDP inhibits the growth of subcutaneous xenografts of MOC-L1

**Fig. 5:**
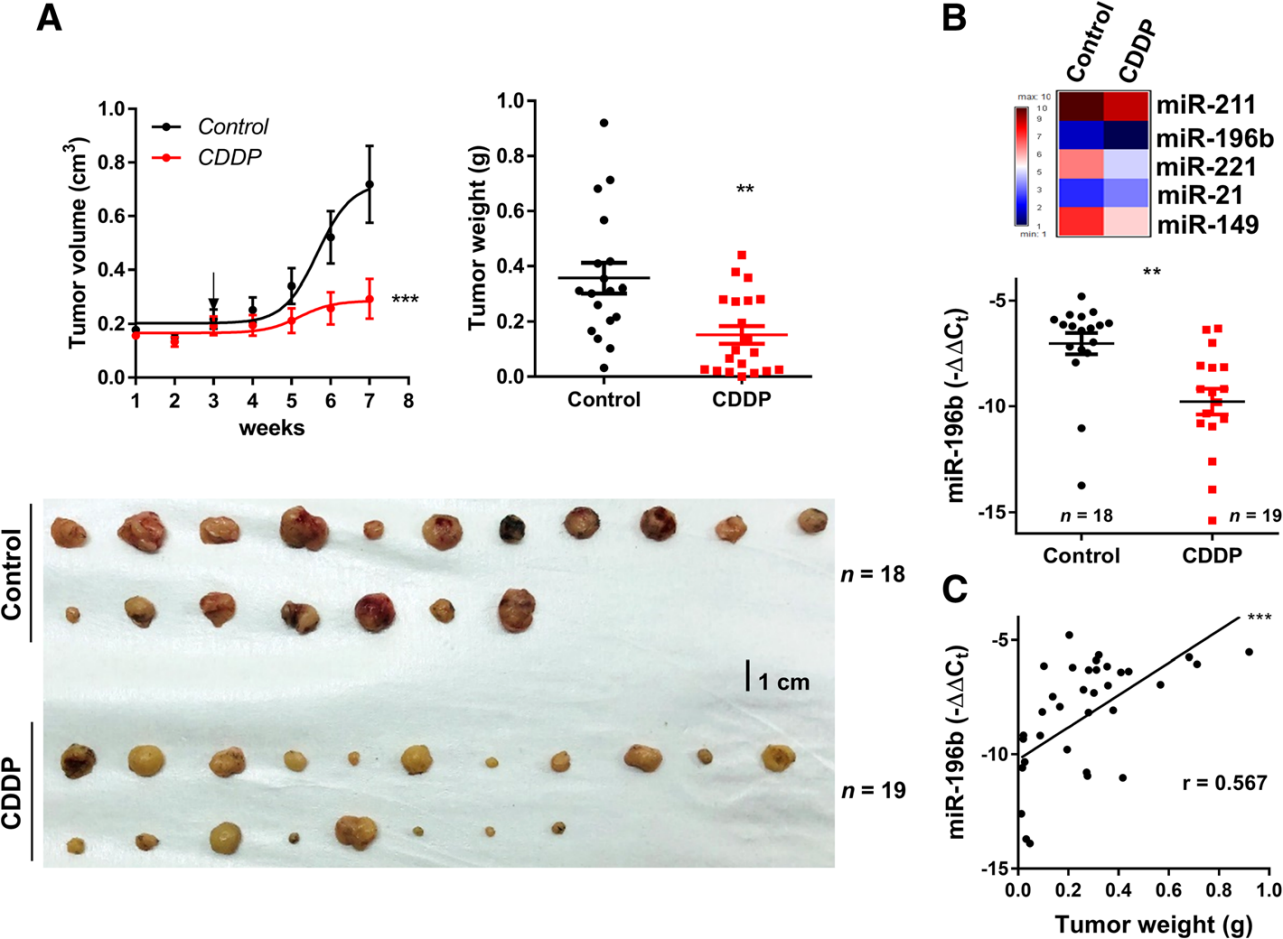
CDDP treatment abrogates the growth of MOC-L1 xenografts. **a** Left, growth curve of subcutaneous MOC-L1 xenografts in C57BL/6 mice over seven weeks. Arrow, start point for intraperitoneally CDDP or normal saline injection. Lower, the resected tumors are harvested at endpoint. Right, quantification of tumor weight. CDDP treatment significantly decreases the weight of the MOC-L1 xenografts. **b** Upper, miRNA expression profile of the control tumors and the CDDP treated tumors. Lower, *miR-196b* expression. The results show a decrease in *miR-196b* expression in the CDDP treated tumors relative to the control tumors. **c** Correlation analysis. This reveals a correlation between *miR-196b* expression and the weight of the xenografts. **, *p* < 0.01; ***, *p* < 0.001

To test the efficacy of CDDP in hampering the subcutaneous growth of MOC-L1, mice bearing xenografts were treated with CDDP intraperitoneally after the tumor had grown to 0.2 mm^3^. This treatment retarded the growth of the tumors and reduced the weight of tumors to about 40% of the control tumors (Fig. 5a). Various miRNAs were highly expressed across all cell lines; however, only miRNAs highly expressed in MOC-L1 or lowly expressed in MOC-L4 were used for this part of the study. The analysis showed that treatment with CDDP resulted in a significant decrease in the expression of *miR-196b*, but not of the other miRNAs in the MOC-L1 tumors (Fig. 5b; Additional file 1: Figure S1b). In addition, the weights of the MOC-L1 xenografts were found to be correlated with the level of *miR-196b* expression (Fig. 5c).

## Discussion

To establish an immunocompetent mouse model is critical to defining pathogenesis, developing preclinical tests and investigating host defense during carcinogenesis. We have successfully established Tg mice lines that exogenously express *miR-31* and *miR-211* in basal keratinocytes. These mouse lines show increased susceptibility to 4NQO induction of tumors due to their defective DNA repair and their oxidative homeostasis [7, 8, 18]. *miR-211* has been reported to be an oncogenic miRNA in OSCC that targets TGF-ẞRII, TCF12 and BIN1, which affects the Myc pathway, antioxidative activation and EGFR/MAPK axis, respectively [7, 8, 15]. Some of these regulatory cascades have been shown to be recapitulated during the oral carcinogenesis process using Tg models [7, 8]. In this study, we established multiple cell lines from the OSCC induced by 4NQO in K14-EGFP-*miR-211* mice. Although these cell lines should have the same transgene background and had been exposed to equivalent doses of 4NQO during tumor induction, their tremendously genetic heterogeneity and their diverse oncogenic properties would seem to reflect the acquisition of different genetic insults during the establishment and progression of the OSCC; this simulates the diversity and complexity of human OSCC cell lines. In general, more than 80% of human *p53* mutations in malignancies are clustered between codons 125 and 300, which corresponds mainly with the DNA binding domain [25]. In addition, more than 40% of HNSCC tumors carry a *p53* mutation. This study identifies three *p53* hotspot mutations involving guanine nucleotides within the four newly established cell lines. Some or all of the *p53* mutations are likely to have been acquired during the 4NQO treatment because 4NQO preferentially targets guanine and therefore the mutation would probably occur before the establishment of these three cell lines [26].

The MOC-L4 cell line shows the weakest oncogenic properties, especially in terms of proliferation and colony formation, which is in agreement with the low expression levels of proliferation, EMT and stemness markers, together with the absence of a *p53* mutation and low level of *miR-196a* expression. By way of contrast, MOC-L1 and MOC-L2 have the potent oncogenic ability, particularly in terms of colony formation and growth in the syngeneic and immune compromised mice lines. These two cell lines show a higher expression of stemness markers compared to MOC-L3 and MOC-L4. MOC-L1 is the most aggressive mouse cell line that we have established. It is characterized by the potent in vitro oncogenicity and in vivo induction of primary xenografts. Relative to MOC-L2, the orthotopic MOC-L1 xenografic tumors give rise to neck nodal metastasis; and on the tail vein injection of MOC-L1, there was extensive lung metastasis and rapid mortality of the syngeneic recipient mice. As a copious induction of EMT markers and stemness markers is present in this cell line, these factors are likely to underlie its high level of aggressiveness. Cancer cell dormancy is thought to be present in tumor progression, which suggests being as a growth arrest and an evolutionary mechanism in metastasis [27]. Whether MOC-L2 is in a dormant state that requires additional factors to activate its metastasis machinery needs specification [28].

Since all of our cell lines are tagged with GFP, this facilitates cell tracing in the lymph nodes and distal organs by fluorescence detection. Furthermore, scattered tumor cells that could be missed during a standard histopathological examination are able to be identified with more certainty by means of fluorescence imaging and/or immunostaining. The fact that this syngeneic mouse model will allow for the profiling of the immune microenvironment, which will help clarifying the disparities between immune-competent mice and immune-compromised mice in neck nodal metastasis. This, in turn, should help addressing the roles of the host defense in counteracting local metastasis, is intriguing. We also further engineered the MOC-L1 cell line to express Luc, which broadens its use in terms of real-time monitoring. Despite the fact that the cell line’s tumorigenic potential is reduced in this cell subclone, its orthotopic growth potential and its ability to produce neck/distal metastases are maintained in this cell subclone. Lingual xenografts that grow to a huge size are prone to induce neck metastasis, but such a primary tumor burden may cause a high mortality risk. As MOC-L1 cell xenografts resulted in high mortality in NOD-SCID mice, we confirmed the local metastasis competence of MOC-L1 cells in nude mice. However, due to the lack of native immune modulation, surveying of immune-escape and evaluation of treatment efficacy do not seem to work well in this system. Therefore, xenotransplantation into other oral sites that will interfere with swallowing to a lesser extent needs to be further developed.

The growth of the subcutaneous xenografts of the MOC-L1 cell line was repressed by CDDP therapy. Analysis indicated that there was a reduced *miR-196b* expression in these tumors relative to untreated tumors. *miR-196b* has been reported to be aberrantly expressed in several cancers [6, 10, 13, 16, 29]. Overexpression of *miR-196b* is commonly found in many digestive tract cancers that have a poor prognosis [6, 29]. It is intriguing to note that there was a decrease in *miR-196b* expression in the CDDP treated mice. These findings seem to suggest that *miR-196b* could be a putative target of CDDP in the MOC-L1 cell line. Furthermore, since expression of *miR-196b* is correlated with the weight of the xenografts, and since *miR-196b* is known to be a circulatory biomarker that can be used for the early detection of OSCC [13], the monitoring of *miR-196b* using body fluids from this immune-competent mouse model could help contributing to marker validation [11].

Although K14-EGFP-*miR-31* Tg mice also exhibited a high susceptibility for 4NQO-induced tongue carcinogenesis [18], the attempts to establish a cell line from the tongue carcinomas of these mice have not succeeded hitherto. However, by crossing K14-EGFP-*miR-31* mice with K14-EGFP-*miR-211* mice, we have generated mouse lines with concordant *miR-31* and *miR-211* overexpression in squamous epithelium. The oral neoplasms spontaneously emerge from these mice would enable the establishment of new cell lines to specify the pathogenic interaction between *miR-31* and *miR-211* [7, 18]. Methylation status, *ETS2* gene and homeobox genes are involved in the regulation of *miR-196b* expression [16, 30, 31]. Since *miR-211* is an oxidative stress inducer [7], and that the oxidation states affect DNA methylation [16], and the expression of *ETS2* and homeobox gene members [32, 33], the potential connection between *miR-211* and *miR-196b* in oral carcinogenesis through such factors remained to be addressed. Furthermore, simultaneous inhibition of both *miR-211* and *miR-196b* to treat OSCC may be a strategy that will help to abrogate OSCC. The roles of *miR-211* in OSCC pathogenesis remain elusive. This study reports four new murine OSCC cell lines exhibiting *miR-211* overexpression. The mechanistic complex of *miR-211*-target gene regulatory axis associating with other genetic alterations, drug responses and oncogenic behaviors can be further specified in these cell models.

The use of cancer organoid lines derived from primary human OSCC can help to predict the therapeutic responses [34]. The usefulness of the syngeneic tumorigenesis models established in preclinical assessment has been defined in a preliminary way. This model should also help to facilitate the therapeutic trials of new drugs. Immunotherapies against OSCC can reduce the toxicities from the conventional radiotherapies and chemotherapies [35]. Till far, most immunotherapeutic approaches on OSCC have been limited in patient studies, because appropriate mouse models that recapitulate the pathological and immunological diversity in human counterparts are lacking. The syngeneic tumorigenesis models established during this study should help to insight tumor immunity and to develop therapeutic strategies.

## Conclusions

In conclusions, this study describes establishing four murine OSCC cell lines from tongue tumor tissue samples, the tumors having been induced by 4NQO using a K14-EGFP-*miR-211* transgenic mouse model. The genetic disruption and aggressiveness of these cell lines, their tumorigenicity and ability to bring about both local regional metastasis and distal metastasis in C57BL/6 syngeneic mice are defined in the present study. These cell lines and the linked immunocompetent animal model that we have established will facilitate the investigation of therapies that can be used to treat OSCC. It seems that exploring the interplay of these OSCC cells with other type of genetically modified cells in vitro or in mice would provide important insights into OSCC pathogenesis.

## Additional file

Additional file 1: **Table S1.** Cell cultivation conditions. **Table S2.** Primers used in the present study. **Table S3.** Primary antibodies or associated reagents used in the present study. **Table S4.** TaqMan^®^ assay probes. **Figure S1.** miRNA expression in the MOC cell lines. **a** An algorithm showing the miRNA expression profiles of MOC-L1 to MOC-L4 cells compared to SAS cells. **b** A comparison of the expression levels of *miR-211*, *miR-196b*, *miR-221*, *miR-149* and *miR-21* between the control tumors and the CDDP treated tumors. *ns*, not significant, **, *p* < 0.01. (DOCX 266 kb)

## Abbreviations

4NQO: 4-nitroquinoline 1-oxide
CDDP: cisplatin
C_t_: Threshold cycle
E-cad: E-cadherin
EMT: Epithelial-mesenchymal transition
GFP: Green fluorescence protein
HNSCC: Head and neck squamous cell carcinoma
IHC: Immunohistochemistry
KSFM: Keratinocyte Serum-Free Medium
MOC-L: mouse oral carcinoma-line
NOD-SCID: Non-obese diabetic CB17-Prkdc^scid^/NcrCrlBltw
OSCC: Oral squamous cell carcinoma
Tg: transgenic
