# Supplementary material for: Establishing of mouse oral carcinoma cell lines derived from transgenic mice and their use as syngeneic tumorigenesis models
Source: BMC Cancer. 2019 Mar 29;19:281. doi: 10.1186/s12885-019-5486-7 (PMC6440159; doi:10.1186/s12885-019-5486-7)
Supplement: Supplementary file 1 — Table S1. Cell cultivation conditions. Table S2. Primers used in the present study. Table S3. Primary antibodies or associated reagents used in the present study. Table S4. TaqMan® assay probes. Figure S1. miRNA expression in the MOC cell lines. a An algorithm showing the miRNA expression profiles of MOC-L1 to MOC-L4 cells compared to SAS cells. b A comparison of the expression levels of miR-211, miR-196b, miR-221, miR-149 and miR-21 between the control tumors and the CDDP treated tumors. ns, not significant, **, p < 0.01. (DOCX 266 kb) [file 12885_2019_5486_MOESM1_ESM.docx]

**Supplementary Tables**

**Additional file 1: Table S1. Cell cultivation conditions**

|  | Culture medium | Supplementary ingredients |
| --- | --- | --- |
| SAS | DMEM | 10% FBS +1% pen-strep-ampho + 2 mM L-glutamine |
| FaDu | MEM | 10% FBS +1% pen-strep-ampho +2 mM L-glutamine |
| MOC-L1~4 | DMEM | 10% FBS +1% pen-strep-ampho + 2 mM L-glutamine |
| 293T | DMEM | 10% FBS +1% pen-strep-ampho |

**Additional file 1: Table S2. Primers used in the present study**

| **Gene or construct** | **Direction** | **Sequences (5’ - 3’)** | **Amplicon (bps)** |
| --- | --- | --- | --- |
| *p53* Exon 1-5 | Forward | CTAGCATTCAGGCCCTCATC | 565 |
|  | Reverse | CTCCGTCATGTGCTGTGACT |  |
| *p53* Exon 5-9 | Forward | TCCCCTCAATAAGCTATTCTGC | 576 |
|  | Reverse | TTCTTTTGCGGGGGAGAG |  |
| *p53* Exon 8-11 | Forward | CAGGGAGCGCAAAGAGAG | 433 or 529 |
|  | Reverse | GGCCAGGAACCACTACTCAG |  |
| Human *PTGER2* | Forward | GCTGCTTCTCATTGTCTCGG | 189 |
|  | Reverse | GCCAGGAGAATGAGGTGGTC |  |
| Mouse *PTGER2* | Forward | CCTGCTGCTTATCGTGGCTG | 189 |
|  | Reverse | GCCAGGAGAATGAGGTGGTC |  |
| Human and mouse *PTGER2* | Forward | TACCTGCAGCTGTACGCCAC | 215 |
|  | Reverse | GCCAGGAGAATGAGGTGGTC |  |

**Additional file 1: Table S3. Primary antibodies or associated reagents used in the present study**

| Antibody | MW (kDa) | Host | Dilution | Supplier | Cat. No. |
| --- | --- | --- | --- | --- | --- |
| GFP | 27 | mouse | 1:1000  1:500# | Clontech | 632381 |
| Ki67 | 345,395 | rabbit | 1:500 | Santa Cruz Biotech | sc7846 |
| PCNA | 34 | mouse | 1:1000 | Santa Cruz Biotech | sc25280 |
| p53 | 53 | rabbit | 1:1000 | Abcam | ab131442 |
| BNC1 | 120 | mouse | 1:1000 | Santa Cruz Biotech | sc517114 |
| Involucrin | 120 | mouse | 1:1000 | Sigma-Aldrich | I9018 |
| TGM1 | 90 | goat | 1:500 | Santa Cruz Biotech | sc18129 |
| K6 | 56 | mouse | 1:1000 | MDBio | LVMS766p0 |
| K14 | 55 | mouse | 1:1000 | Abcam | ab7800 |
| K18 | 45 | mouse | 1:1000 | Santa Cruz Biotech | sc32329 |
| K19 | 40 | mouse | 1:1000 | Santa Cruz Biotech | sc376126 |
| E-cadherin | 120 | mouse | 1:1000 | BD | 610182 |
| Vimentin | 57 | rabbit | 1:1000 | Cell Signaling | 5741 |
| Fibronectin | 240 | mouse | 1:1000 | BD | 610078 |
| Twist | 17,26 | rabbit | 1:1000 | Santa Cruz Biotech | sc15393 |
| ZEB1 | 200 | rabbit | 1:1000 | Cell Signaling | 3396S |
| αγSMA | 43 | mouse | 1:1000 | Santa Cruz Biotech | sc53015 |
| αV Integrin | 135,140 | rabbit | 1:1000 | Cell Signaling | 4711 |
| MMP2 | 63,72,89 | mouse | 1:1000 | Santa Cruz Biotech | sc13595 |
| MMP9 | 105 | rabbit | 1:1000 | Sigma-Aldrich | M9555 |
| SOX2 | 35 | rabbit | 1:1000 | Cell Signaling | 3579S |
| Nanog | 42 | rabbit | 1:1000 | Cell Signaling | 3580S |
| Oct4 | 45 | rabbit | 1:1000 | Cell Signaling | 2750S |
| GAPDH | 36 | mouse | 1:10000 | Santa Cruz Biotech | sc32233 |

#, concentration used for IHC

**Additional file 1: Table S4. TaqMan^®^ assay probes**

| Gene and miRNA | Cat. No. |
| --- | --- |
| *mmu-miR-31* | 000185 |
| *miR-16* | 000391 |
| *miR-19a* | 000395 |
| *miR-21* | 000397 |
| *miR-24* | 000402 |
| *miR-27a* | 000408 |
| *miR-27b* | 000409 |
| *miR-34c* | 000428 |
| *miR-103* | 000439 |
| *miR-107* | 000443 |
| *miR-125b* | 000449 |
| *miR-132* | 000457 |
| *miR-134* | 000459 |
| *miR-146a* | 000468 |
| *miR-181a* | 000480 |
| *miR-181c* | 000482 |
| *miR-184* | 000485 |
| *miR-194* | 000493 |
| *miR-210* | 000512 |
| *miR-221* | 000524 |
| *miR-20a* | 000580 |
| *mmu-miR-211* | 001199 |
| *U6 snRNA* | 001973 |
| *miR-29a* | 002112 |
| *miR-196b* | 002215 |
| *miR-149* | 002255 |
| *miR-370* | 002275 |
| *miR-145* | 002278 |
| *miR-200c* | 002300 |
| *miR-17* | 002308 |
| *miR-543* | 002376 |
| *miR-144* | 002676 |

**Supplementary Figures**

**
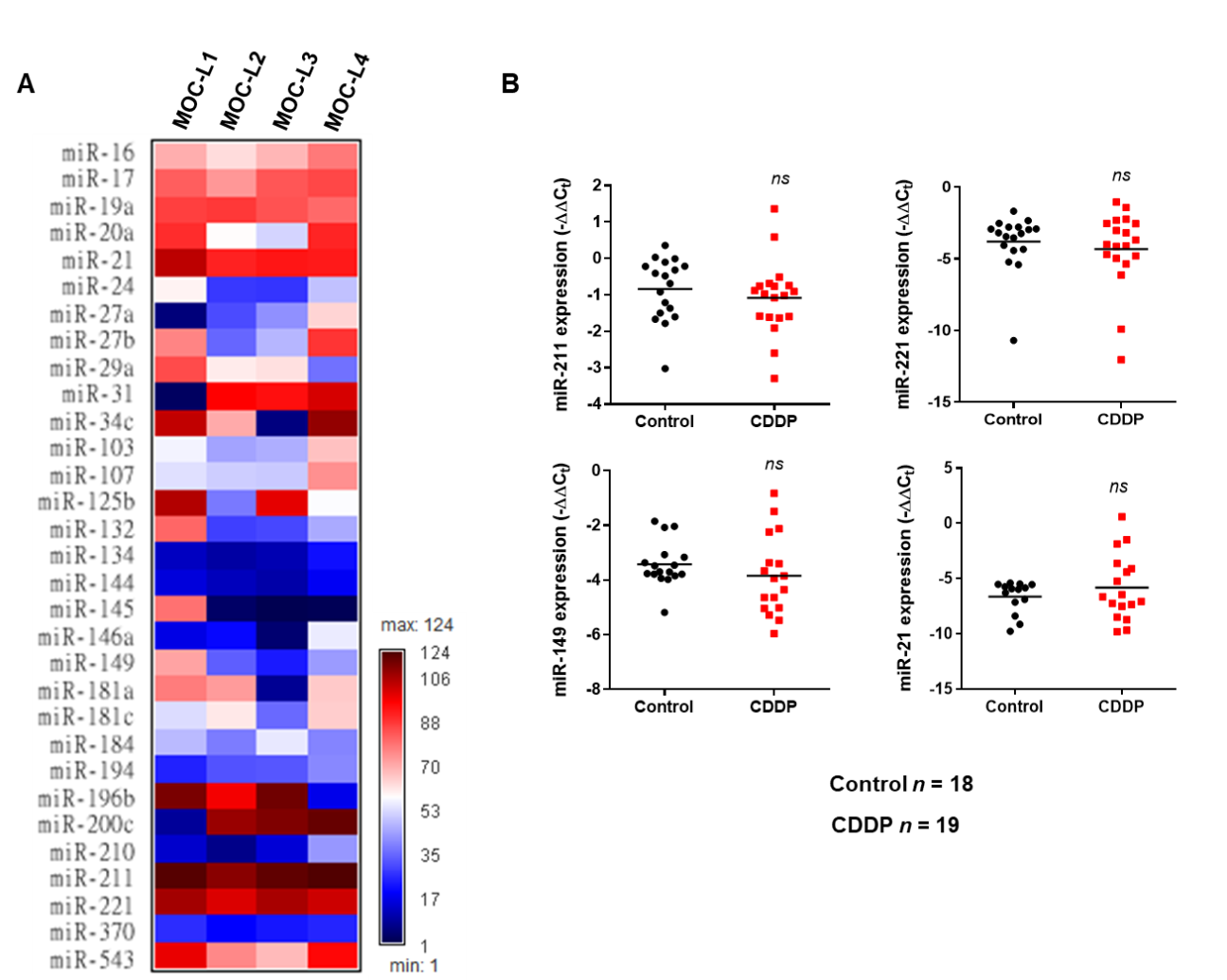
**

**Additional file 1: Figure S1. miRNA expression in the MOC cell lines.** (A) An algorithm showing the miRNA expression profiles of MOC-L1 to MOC-L4 cells compared to SAS cells. (B) A comparison of the expression levels of *miR-211*, *miR-196b*, *miR-221*, *miR-149* and *miR-21* between the control tumors and the CDDP treated tumors. *ns*, not significant, **, *p* < 0.01.
